# Post-9/11 Veterans and Their Partners Improve Mental Health Outcomes with a Self-directed Mobile and Web-based Wellness Training Program: A Randomized Controlled Trial

**DOI:** 10.2196/jmir.5800

**Published:** 2016-09-27

**Authors:** Janet R Kahn, William Collinge, Robert Soltysik

**Affiliations:** ^1^ College of Medicine Department of Psychiatry University of Vermont Burlington, VT United States; ^2^ Collinge and Associates, Inc. Eugene, OR United States; ^3^ Optimal Data Analysis, LLC Glen Burnie, MD United States

**Keywords:** veterans, PTSD, moral injury, mind-body therapies, mindfulness, patient-centered care, compassion, Web-based program, reintegration

## Abstract

**Background:**

Veterans with history of deployment in the Global War on Terror face significant and ongoing challenges with high prevalences of adverse psychological, physical, spiritual, and family impacts. Together, these challenges contribute to an emerging public health crisis likely to extend well into the future. Innovative approaches are needed that reach veterans and their family members with strategies they can employ over time in their daily lives to promote improved adjustment and well-being.

**Objective:**

The objective of this study was to evaluate effects of use of a Web-based, self-directed program of instruction in mind- and body-based wellness skills to be employed by Global War on Terror veterans and their significant relationship partners on mental health and wellness outcomes associated with postdeployment readjustment.

**Methods:**

We recruited 160 veteran-partner dyads in 4 regions of the United States (San Diego, CA; Dallas, TX; Fayetteville, NC; and New York, NY) through publicity by the Iraq and Afghanistan Veterans of America to its membership. Dyads were randomly allocated to 1 of 4 study arms: Mission Reconnect (MR) program alone, MR plus the Prevention and Relationship Enhancement Program (PREP) for Strong Bonds weekend program for military couples, PREP alone, and waitlist control. We administered a battery of standardized and investigator-generated instruments assessing mental health outcomes at baseline, 8 weeks, and 16 weeks. Dyads in the MR arms were provided Web-based and mobile app video and audio instruction in a set of mindfulness-related stress reduction and contemplative practices, as well as partner massage for reciprocal use. All participants provided weekly reports on frequency and duration of self-care practices for the first 8 weeks, and at 16 weeks.

**Results:**

During the first 8-week reporting period, veterans and partners assigned to MR arms used some aspect of the program a mean of 20 times per week, totaling nearly 2.5 hours per week, with only modest declines in use at 16 weeks. Significant improvements were seen at 8 and 16 weeks in measures of posttraumatic stress disorder, depression, sleep quality, perceived stress, resilience, self-compassion, and pain for participants assigned to MR arms. In addition, significant reductions in self-reported levels of pain, tension, irritability, anxiety, and depression were associated with use of partner massage.

**Conclusions:**

Both veterans and partners were able to learn and make sustained use of a range of wellness practices taught in the MR program. Home-based, self-directed interventions may be of particular service to veterans who are distant from, averse to, or prohibited by schedule from using professional services. Leveraging the partner relationship may enhance sustained use of self-directed interventions for this population. Use of the MR program appears to be an accessible, low-cost approach that supports well-being and reduces multiple symptoms among post-9/11 veterans and their partners.

**Trial Registration:**

Clinicaltrials.gov NCT01680419; https://clinicaltrials.gov/ct2/show/NCT01680419 (Archived by WebCite at http://www.webcitation.org/6jJuadfzj)

## Introduction

Since 2001, roughly 2.3 million US military personnel have been deployed to the Global War on Terror, many more than once. These veterans have displayed high rates of comorbidity of chronic pain, posttraumatic stress disorder (PTSD), mild traumatic brain injury, and other conditions, creating an urgent need for innovative, accessible interventions and multimodal treatment approaches [1,2]. PTSD rates are estimated at ≤30% [3]. A review of 29 studies found “prevalence rates of adult men and women previously deployed ranging from 5% to 20% for those who do not seek treatment and around 50% for those who do seek treatment” [4].

In addition, up to 81.5% of Global War on Terror veterans have acute or chronic pain [5-7]. In fact, many veterans live with *complex pain* resulting from multiple physical injuries, the pain from which may be exacerbated by high rates of emotional distress and mental problems resulting from traumatic brain injury [8]. Suicidality also remains a concern. A recent analysis of the military suicide prevention provisions mandated by a presidential executive order in 2012 concludes they have not been fully and effectively implemented and the goal of reducing military suicide “remains elusive” [9].

Postdeployment screening has suggested that many returning veterans may have problems that warrant treatment, but the majority may not receive treatment [10,11]. The RAND Center for Military Health Policy Research found that barriers deterring veterans from seeking help include concerns about negative career repercussions, belief that treatment won’t be effective, the prospect of long wait times, limited availability of providers, and the potential side effects of medications. They concluded that continued research is needed to develop more effective treatment options [12]. Together, the complexity of many veterans’ situations and the multiple interferences with receiving treatment contribute to an emerging public health crisis that is likely to extend well into the future [13].

While veterans face several risk factors for long-term mental health problems, higher interpersonal support is protective [14]. The critical role of such support was recognized by a joint work group of researchers from the US National Institute of Mental Health, the US Department of Veterans Affairs (VA), and the US Department of Defense (DoD) that singled out couple-focused interventions as among the needed directions for new research [15]. Indeed, significant attention has been focused on the impact of deployment on the spouse, as 55% of returning soldiers are married [16]. Evidence indicates that spouses may experience greater levels of emotional stress than soldiers, with soldiers’ combat exposure reflected in higher spousal stress levels [17]. A review of 14 studies found that longer deployments, deployment extensions, and PTSD in military personnel were associated with psychological problems for the spouse [18]. Finally, there is evidence that the stresses of deployment may adversely affect marital satisfaction in military couples well after a return [19]. Thus, for a primary relationship to serve the much-needed support function for veterans, the impact of deployment on the *veteran*, the *partner*, and the *relationship* all need to be recognized and addressed.

The DoD’s most widely used effort to engage the relationship dyad in reintegration has been the Prevention and Relationship Enhancement Program (PREP) for Strong Bonds (PREP Inc, Greenwood Village, CO, USA), a standardized program administered by military chaplains [20]. However, due to limited resources, the Strong Bonds program has the modest goal to target only 18% of postdeployment Army personnel and their families (Chaplain (Maj) J Bartels, Strong Bonds Program Operations Manager, Chaplaincy Headquarters, US Department of the Army, oral communication, June 8, 2016). Additional smaller efforts exist, such as Families OverComing Under Stress [21], and most recently the VA system has begun offering the Warrior to Soulmate program at a few VA hospitals [22]. Unfortunately, such programs have inherent barriers limiting their reach into the full population in need. These include geographic distance, required time away from work and children, a group-based experience, which is aversive to some, and the requirement of qualified professional leadership, which is costly and not always readily available.

Alternative approaches are needed that are both accessible and acceptable to veterans and their partners, to help mitigate the long-term impacts of deployment on their well-being and relationship stability. One such approach is the use of Internet-based multimedia instruction in both individual and collaborative self-care strategies. This paper reports the results of a study of an integrated program of mind- and body-based therapies delivered in multimedia format by the Internet and mobile app. Entitled Mission Reconnect (MR), the program was designed as a dyadic intervention for post-9/11 veterans and their partners to use individually and together, teaching selected self-care strategies aimed at addressing short- and long-term impacts of deployment and promoting well-being.

A phase I feasibility study of MR found high compliance and significant improvements in measures of perceived stress, depression, PTSD, and self-compassion for both members of the dyad. In addition, significant reductions were reported in pain, tension, irritability, anxiety, and depression for veterans following partner-delivered massage [23]. These results indicated that meaningful improvements in well-being are possible with this form of intervention. The objective of this subsequent study is to evaluate effects of use of MR on mental health outcomes associated with postdeployment readjustment in a randomized controlled trial with an active comparator (ClinicalTrials trial registration number NCT01680419).

## Methods

### The MR Program

The MR program is grounded in the biopsychosocial model of health [24]. Just as the risks and threats to well-being in the target population are multidimensional and affect multiple systems (psychological, social, spiritual and physical), multidimensional intervention addressing these multiple systems simultaneously may be beneficial. VA staff treating this comorbid population have coined the term postdeployment multisymptom disorder and noted that treatments focusing on only a single condition or symptom produce “suboptimal outcomes” [25]. Thus, we composed an integrated program of practices supporting psychological (PTSD, stress, depression, resilience, self-compassion), social (mutual support, relationship satisfaction, collaborative participation, compassion), and physical outcomes (physical pain, tension, sleep quality).

The specific methods used are grounded in the evidence bases of mindfulness-based therapies [26,27], massage therapy [28,29], positive emotions [30-32], and caregiver education [33,34]. MR is designed for autonomous, self-directed use without dependence on professional instruction. This makes it a resource for users not accessing formal services, whether due to geographic obstacles, lack of suitable services, or personal preference. It is not designed as a substitute for formal clinical services but can be used to complement or enhance them.

The program delivers the instruction by techniques grouped into three categories— *connecting with yourself*, *connecting with quiet*, and *connecting with your partner* —together comprising the 11 individual activities described in Table 1. Instruction is provided via videos, guided audio exercises, and written materials, all accessible on the program website [35] and mobile device apps (iOS, Android, and Windows Phone versions). The study website was a static design but has been a made responsive design since completion of the study. The information offered on the website and the mobile app is redundant except for the print materials (see the Supporting Materials subsection below), which can be downloaded from the website only.

#### Video Content

**Figure 1:**
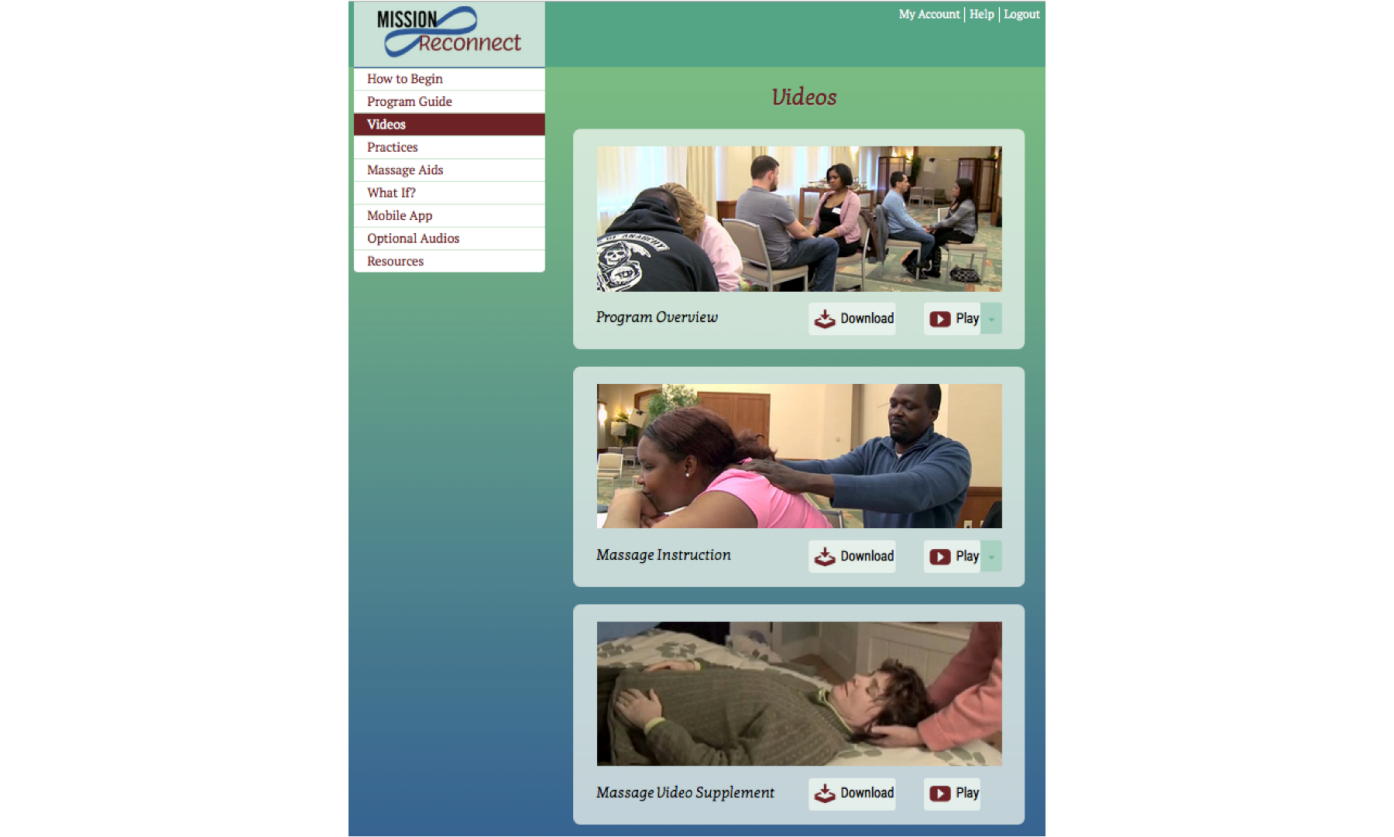
Mission Reconnect Video Menu.

We obtained instructional footage by filming 2 workshops teaching MR to 11 Iraq and Afghanistan veterans and their partners. The Program Overview video (54 minutes) introduces the program and provides instructional sequences for the individual exercises, accompanied by commentary and discussion by workshop participants and Dr Wayne Jonas, a former military physician and expert in the field of integrative health care. Footage of massage instruction, also with veterans and partners, is presented in the program’s Massage Instruction video (37 minutes). A Massage Video Supplement (3 minutes) addresses logistics of using home furniture. The video menu, as participants saw it, can be viewed in Figure 1.

#### Audio Content

**Figure 2:**
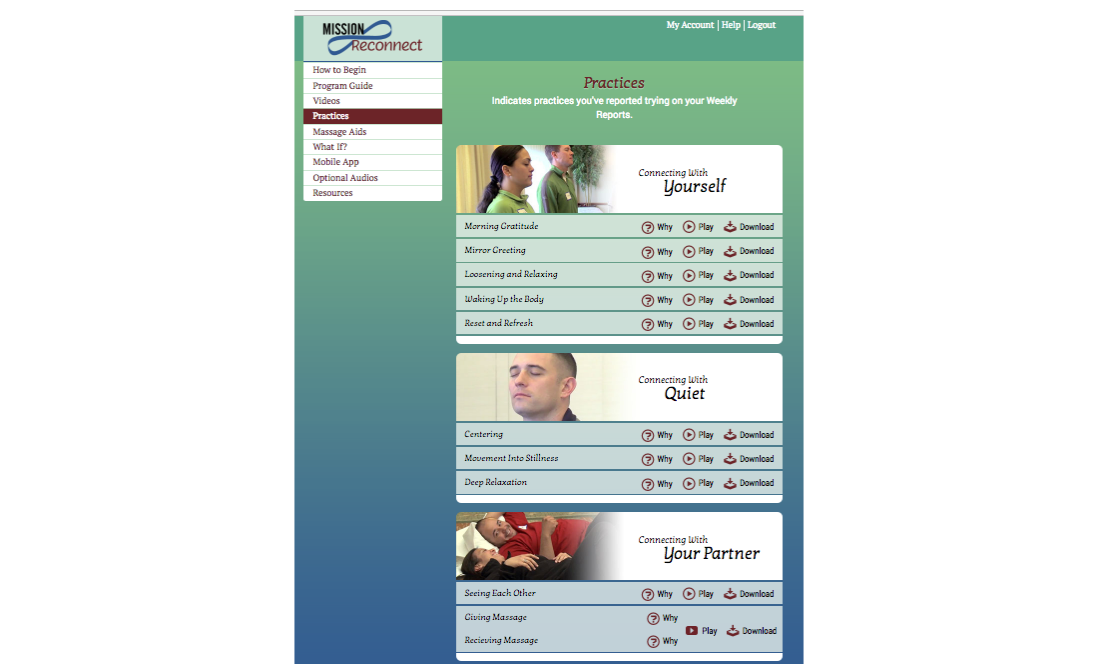
Mission Reconnect Practice Menu.

The 9 audio exercises range in length from 1 to 22 minutes. Users are encouraged to listen, learn the practices, and then use each technique with or without guided instruction as they wish. As Figure 2 indicates, users could also view reasons why to use each practice. Clicking on Why for any practice would produce 3 to 5 brief statements, based on research, indicating potential benefit from that practice. For instance, for centering, they would see “Helps calm and relax the mind and body;” “Helps you feel grounded and present;” “Helps you be less reactive to thoughts and feelings;” “Gives you more choice about how to respond to events and feelings;” and “Helps you feel peaceful more easily.”

#### Supporting Materials

**Figure 3:**
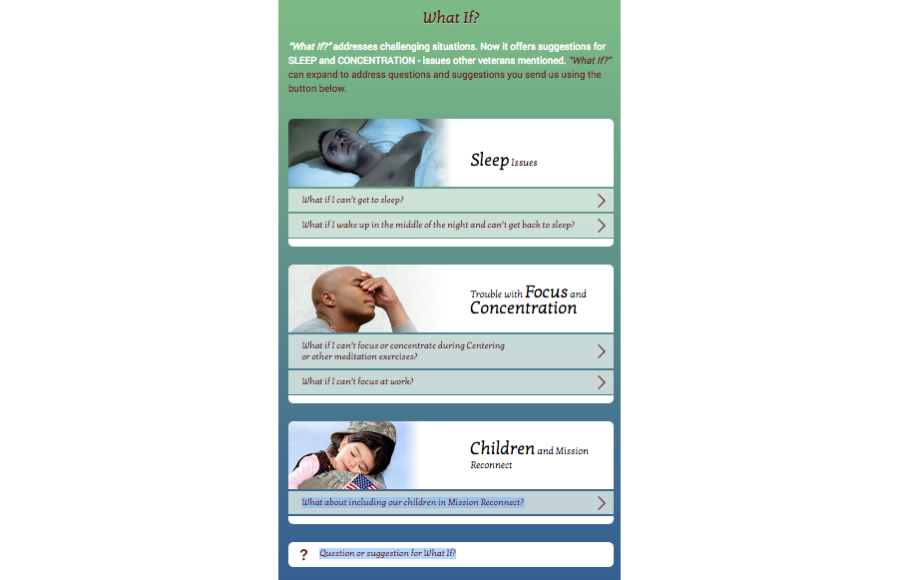
Mission Reconnect What If? Menu.

A massage instruction booklet and a 1-page illustrated massage reminder handout are downloadable from the website [35]. A *What if?* feature on the website and the app enables users to access advice on how to apply program techniques in specific challenging situations such as problems with sleep, focus, and concentration. Clicking on any of the arrows next to a *What If?* topic, as seen in Figure 3, would open to a page displaying both written suggestions and active audio links. Users can submit questions through the *What if?* interface or suggest future content to enhance the program. *Optional Audios* include the guided audio exercises using the alternative gender voice to that used in the main program (see Multimedia Appendix 1 for a video introduction to MR offering an overview of the elements of the program).

**Table 1:** Content of the Mission Reconnect Program.

Component		Description	Run time	Media
Program overview		Introduction to the structure and components of the program; instructional sequences; motivational interviews with experts and participants.	54:29	Video
**Connecting with yourself**							
	Morning gratitude	A brief practice of starting each day with a moment of spoken gratitude to encourage positive mood, empathy, forgiveness, focus, and sleep quality.	2:49	Audio
	Mirror greeting	A brief practice of greeting oneself in a mirror with positive self-regard to encourage self-confidence and resilience, and to decrease tension.	1:18	Audio
	Loosening and relaxing	A brief practice of standing and rhythmically moving to relax, release physical tension, bring awareness into the body, and feel happier.	5:50	Video & audio
	Waking up the body	A brief practice of rhythmically patting and stimulating meridians to increase energy and blood flow, and one’s sense of aliveness.	9:26	Video & audio
	Reset and refresh	Evoking a series of yawns to release tension, interrupt stressful patterns of thought or feeling, prompt oxytocin production, and return attention to the present moment.	2:42	Video & audio
**Connecting with quiet**							
	Centering	A guided exercise of basic mindfulness instruction focused on using the breath as an anchor to the present moment and to reduce reactivity.	9:41	Video & audio
	Movement into stillness	A brief practice of gentle swaying movement to bring body and mind into alignment in the present moment. Effects are similar to centering; method is ideal for those who cannot sit still.	4:08	Video & audio
	Deep relaxation	A guided exercise lying down or sitting and combining aspects of traditional progressive relaxation and yoga nidra meditation, for sleep enhancement and relaxation.	23:12	Video & audio
**Connecting with your partner**							
	Seeing each other	A guided contemplative exercise focused on eliciting appreciation, compassion, and forgiveness for one’s partner and oneself, done alone or together.	8:00	Video & audio
	Giving massage Receiving massage	Practicing being present with one’s partner by providing comfort and relaxation through simple massage techniques, and by receiving massage.	37:35 main, 3:00 supplement, booklet 34 pp, reminder handout 2 pp.	Video & print

### Aims and Hypotheses

Aim 1 was to evaluate the impact of MR on mental health outcomes associated with postdeployment readjustment (primary outcomes). Hypothesis 1 was that veterans and partners assigned to MR would report significantly greater improvements in stress, depression, PTSD symptoms, self-compassion, sleep quality, resilience, social support, and relationship satisfaction compared with those in the non-MR comparison groups.

Aim 2 was to evaluate program use and satisfaction among participants in the MR program (secondary outcomes). Hypothesis 2 was that participants assigned to MR would comply with recommendations to use the nonmassage techniques of the program weekly and would average 30 minutes per week of nonmassage practice. Hypothesis 3 was that participants assigned to MR would comply with recommendations to exchange massages weekly and would receive an average of at least one massage per week. Hypothesis 4 was that users of MR receiving massage would report significant positive massage effects.

Aim 3 was to collect exploratory data on change in pain levels associated with the use of MR. There were no specific hypotheses associated with this aim.

Aim 4 was to collect exploratory data on the presence of moral injury. There were no specific hypotheses associated with this aim.

### Study Design

To evaluate outcomes of MR, we planned a 4-arm randomized controlled trial using a standard-of-care comparator to evaluate both comparative and additive effects over a 16-week period. The comparator was the PREP for Strong Bonds program used widely by the military for relationship enhancement and postdeployment reintegration [36]. This is a standardized, evidence-based program conducted during weekend residential retreats with experiential exercises, facilitated by Army Chaplain Corps-trained chaplains. The multidimensional program uses methods from cognitive behavioral therapy and communication-oriented marital enhancement programs developed by Markman et al, Denver University Center for Marital and Family Studies [37]. Thus, the 4 arms to be studied were (1) MR alone, (2) MR+PREP, (3) PREP alone, and (3) a waitlist control. Sample size was determined by power analysis applied to the phase I feasibility data [23]. We planned a sample of 160 dyads, with 40 dyads allocated to each arm.

#### Participants

##### Eligibility

Eligible applicants were veteran-partner dyads in which the veteran had a history of deployment in a post-9/11 combat operation. This includes what the US DoD has labeled Operation Iraqi Freedom, Operation Enduring Freedom, and Operation New Dawn. As proof of deployment, veterans typically provided their Form DD214, a discharge or separation from service document provided by the military that includes the veteran’s military service record. This verification of deployment had to be provided before random allocation. The partner could be a spouse, life partner, fiancé or fiancée, girlfriend or boyfriend, or in other significant relationship identified by the veteran as fulfilling the role of “partner” for the purposes of mutual support. While most dyads had partners with no military experience, some dual-veteran dyads also applied and were accepted. No queries were made regarding prior use of meditation or other complementary and alternative techniques.

##### Publicity and Recruitment

Iraq and Afghanistan Veterans of America provided publicity for recruitment through their website, social media channels, and geographically targeted emails to their membership announcing the study [38]. To achieve geographic diversity, we recruited study cohorts for all 4 study arms in each of 4 metropolitan areas: San Diego, CA; Dallas, TX; Fayetteville, NC; and New York, NY, USA.

##### Application and Consent

The New England Independent Review Board (Needham, MA) provided institutional review board oversight. Prospective participants completed a Web-based application (PsychData, State College, PA, USA), which included the consent form (downloadable as a pdf file). Applicants gave electronic consent by marking a box attesting they had read the consent form when they submitted the application. Applicants were interviewed by phone by a research assistant to determine candidacy—that is, that the dyad met all the eligibility requirements and was available for the planned meeting date in their city if they were selected. Launch meetings were held in each city for each arm (content is listed below in the Protocols subsection). All meetings were held in hotel conference rooms, except in New York, where the MR-only and waitlist meetings were held in a conference room at the offices of Iraq and Afghanistan Veterans of America, the organization that had helped in recruitment.

##### Random Allocation

From each city’s eligible candidate pool, we selected, based on our ethnic and racial diversity criteria, 40 candidate dyads to be randomly allocated to the 4 arms in that city (random allocation was generated through Randomization.com [39]). Candidates not selected were placed on an alternates list. After random allocation and before any data collection, we notified selected dyads of their assignment and required date of attendance at a launch meeting to begin the study for their arm. Those who declined or dropped out before the launch meeting were replaced by a randomly selected dyad from that city’s alternates list, to fill the vacated slot so as to preserve the original randomization. If a city had insufficient candidates to fill a slot, we moved the empty slot to its same arm in another city and used the new city’s alternates list to randomly select a dyad for the vacant slot, to satisfy the overall study randomization plan of 40 dyads per arm (this was done twice).

#### Protocols

##### MR-Only

Dyads attended a 90-minute launch meeting designed to ensure that they understood their responsibilities to the study, and the logistics of both data collection and participant compensation. Each MR-only launch meeting included all dyads in that city that had been randomly allocated to the MR-only arm. The investigators introduced themselves, attendees introduced themselves, and the investigators went over the logistics of participation. The 10-minute Introduction chapter of the Program Overview video was shown followed by explanation of data collection procedures materials. Participants’ responsibilities were described, including trying all MR techniques at least once during the first few weeks, sharing weekly massage with one’s partner, and providing Web-based data through 8 weekly reports and 3 surveys. Data collection procedures were explained, as were the mechanics of compensation linked to data collection, and each participant received a debit card that had been preloaded with compensation for the baseline survey they had completed prior to the launch meeting. The importance to the study of the MR-only arm was explained, participants were thanked for their willingness to participate in the study, and the meeting was concluded. No actual instruction or practice took place in the launch meeting. For the duration of the study, MR dyads received a weekly e-newsletter from the investigators focused on general reiteration of instructions and encouragement to try all the practices.

##### MR+PREP

Dyads attended a standardized weekend residential PREP retreat led by a PREP-trained army chaplain. A total of 3 chaplains were used across the 4 cities. A teleconference among the chaplains, investigators, and a trainer from PREP Inc headquarters was conducted to establish fidelity with current PREP content, and a standard program with 12 hours of content was agreed upon before the study. PREP weekends lasted from Friday evening to noon Sunday. Instruction focused on communication and relationship building, problem solving, stress and relaxation, intimacy, forgiveness, and commitment. After lunch Sunday, the dyads attended an MR launch meeting with the same protocol as described for the MR-only participants above.

##### PREP-Only

Dyads attended a standardized PREP weekend as described above. At the program’s conclusion, the chaplain (scripted by the investigators to be the same as for waitlist participants, described below) provided the launch meeting content for non-MR participants. As with other arms, attendees were given instructions for data collection on the project website for the remainder of the study. The participants’ compensation was explained and attendees received their debit cards that had been preloaded with compensation for the baseline survey they had completed prior to the PREP weekend. The importance of their arm to the integrity and value of the study was explained, and participants were thanked for their willingness to participate in the study. Participants were instructed to continue their usual behavior regarding self-care or wellness-related activity. At the end of the study, they would be given access to the MR program.

##### Waitlist

Dyads attended a 90-minute launch meeting with the investigators to receive their instructions for data collection on the project website. As with other arms, they were introduced to the purpose of the project, the design of the study, the mechanism for their compensation, and the importance of the waitlist control arm to the study. They were instructed to continue with their usual behavior regarding self-care or wellness-related activity. They were thanked for their participation and given their debit cards preloaded with compensation for the baseline survey they had completed prior to the meeting. At the end of the study, they would be given access to the MR program.

#### Human Contact at Launch

We recognized in designing the study that the quantity of human contact at launch for each arm was an important consideration; hence, we planned to assure that the MR-only and waitlist arms had an equal duration (90 minutes) of contact during launch. The 2 PREP arms of course received a full weekend intervention in person to allow the comparisons between self-directed and in-person intervention sought in the study. Between the 2 PREP arms, there was some difference in total contact over the full weekend, with the PREP-only group receiving less time dedicated to launch information because there was no need for the MR portion. We deemed this difference of negligible importance given participants’ exposure to a full weekend of contact; it proved most practical to incorporate their launch content at the end of their workshop rather than making them stay an extra 90 minutes.

#### Prompting

Each participant received email notifications of data collection tasks due (weekly reports, surveys). A personal project calendar on the website and app displayed her or his data collection due dates (non-MR participants could not access intervention content).

#### Data Collection

All data collection was Web based. The initial application was submitted on PsychData.com, and surveys and weekly reports during the study period were completed on the project website.

##### Survey Data

A survey package was administered 3 times: at baseline (T1, prior to the launch meeting), at 8 weeks (T2), and at 16 weeks (T3, end of study). We used the following standardized instruments: the Perceived Stress Scale-10 item (PSS) [40], Beck Depression Inventory (BDI) [41], PTSD Checklist-Civilian version (PCL-C) [42], Self-Compassion Scale (SCStotal) [43], Response to Stressful Experiences Scale (RSES) [44], Multidimensional Scale of Perceived Social Support (MSPSStotal) [45], Pittsburgh Sleep Quality Index (PSQItotal) [46], and Revised Dyadic Adjustment Scale (RDAS) [47]. In addition, 2 investigator-generated Likert-scaled (0–10 points) questions asked respondents to rate their usual pain level over the past week (PainUsual) and their best pain level over the past week (PainBest).

Veterans were asked 5 investigator-generated items exploring the concept of moral injury associated with military service. The results of this exploratory moral injury inquiry are not reported here, as addressing moral injury directly was not an aim of the MR program. We will report those data in a subsequent publication.

##### Weekly Reports

During the initial 8 weeks, all participants completed a Web-based weekly report on their use of wellness activities as follows (see Multimedia Appendix 2 and 3 for weekly report forms used by MR and non-MR participants).

For the MR arms, the weekly report recorded frequency and duration of use of each MR program activity. In addition, participants were to designate one 20-minute massage per week as their massage reporting session, for which they completed a massage session card (hard copies were provided at the launch meeting). On the card, they rated symptoms of pain, tension, being on edge or irritable, anxiety or worry, and depression, on a 0–10 Likert scale, both before and again 15 minutes after the massage (data on massage session effects). They were then to upload the responses recorded on the card to their weekly report.

For participants in the non-MR arms, the weekly Web-based report assessed the number and types of activities used during the past week to (1) relax, reduce stress, or support general well-being, (2) ease physical pain or tension, (3) support one’s partner or strengthen the relationship, and (4) improve sleep quality.

No weekly reports were collected during weeks 9–15 of the study. In the final survey package (16 weeks), a weekly report was included for the last week of activity.

##### Compensation

Each participant was given a debit card at their launch meeting for payment for data collection. Cards were automatically funded via the data collection website when reports and surveys were submitted: US $40 after each survey and US $20 after each weekly report.

### Statistical Methods

We used intent-to-treat analysis. For the survey data, paired-sample *t* tests were performed for each pairwise contrast of times T1 (baseline), T2 (8 weeks), and T3 (16 weeks). Descriptive statistics were used to report the weekly report data for MR arms and non-MR arms. Pre-post massage session effects were evaluated using the Wilcoxon signed rank test for each weekly pairwise contrast. In addition, Kendall tau-b was evaluated across the 8-week set of samples of premassage effects. The 4 study arms were compared with each other for each of the survey outcomes at each time point using 2-sample *t* tests. The MR and MR+PREP groups were combined to define a common metric concerning frequency and duration of MR activities. This metric was correlated to the survey data and analyzed by Pearson correlation. Improvement in survey scores at time T2 was also predicted from the frequency and use of MR activities with Kendall tau-b. Linear discriminant analysis was performed at baseline to investigate differences in survey outcomes at T2 and T3 between the waitlist control and each of the other 3 comparison groups.

We used a sequentially rejective Sidak Bonferroni-type multiple comparisons procedure to ensure the desired experimentwise type I error rate (*P*<.05). This procedure has been demonstrated to more efficiently ensure the desired experimentwise *P* value when compared with Dunn’s procedure [48].

For dual-veteran couples, we performed subanalyses for veterans as a class and nonveteran partners as a class. In dyads where both members met the criteria for veteran, we included both in analyses of veteran data, and not in partner data.

## Results

### Sample

**Figure 4:**
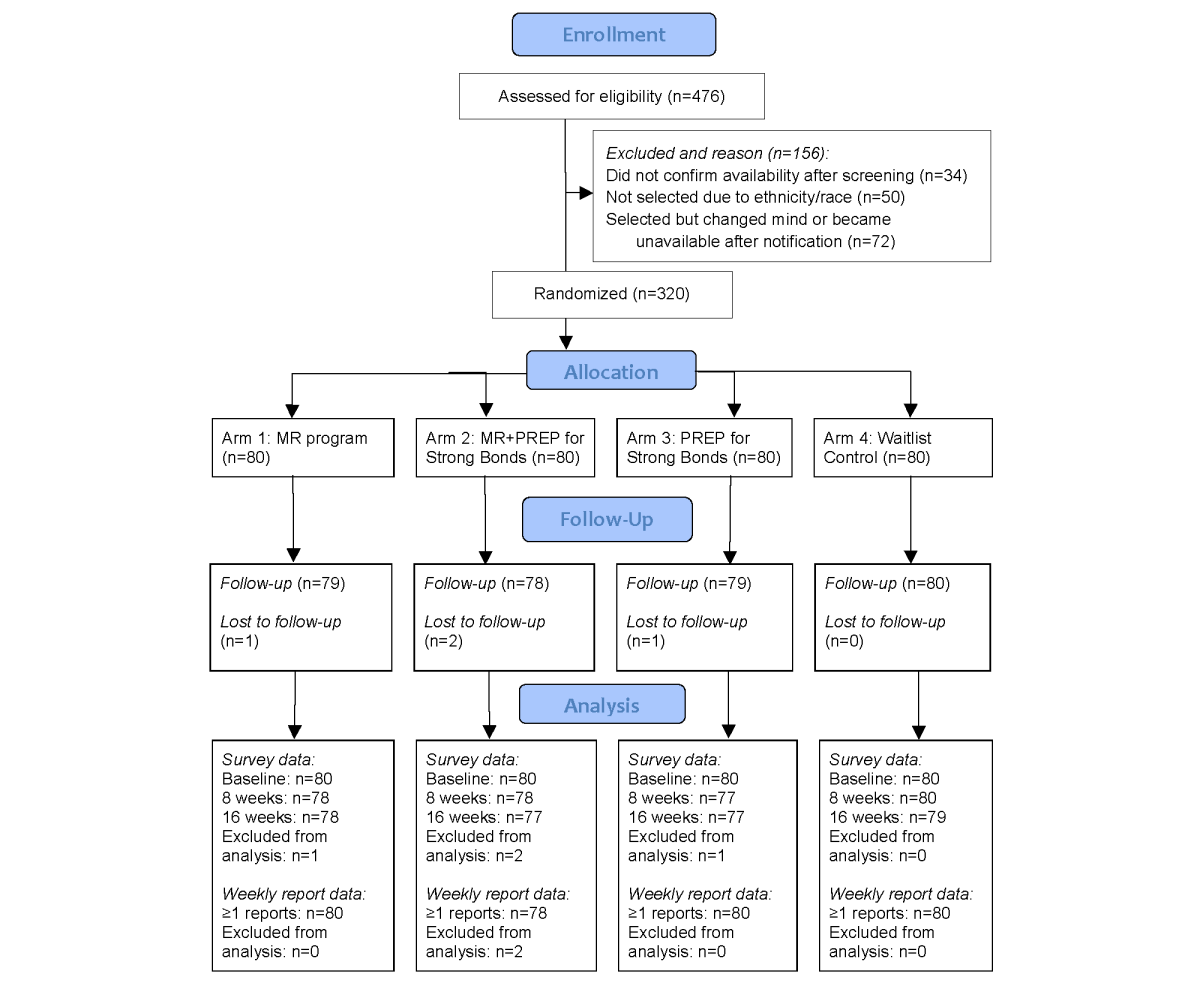
CONSORT Flow Diagram.

A total of 238 dyads (476 individuals) that met eligibility criteria applied and were assessed for candidacy for 160 slots. This process included submission of a copy of a DD214 form or other official military document that verified their deployment status. Figure 4 shows the reasons prompting exclusion from selection and the flow of participants through the project (see Multimedia Appendix 4 [49] for the CONSORT eHealth checklist). All 320 participants completed their baseline surveys, with 313 completing the 8-week follow-up and 311 also completing the 16-week follow-up. Within the sample of 160 dyads were 21 dyads in which both members were veterans. Thus, the sample comprised 181 veterans and 139 nonveteran partners (hence the differences in numbers in the tables below separately reporting veteran and partner outcomes).

#### Demographics

Most dyads (151/160, 94.4%) were married or living together as life partners. Mean relationship duration was 7.2 years with a range of 1–32 years. The sample included 3 same-sex couples and 21 couples in which both members met the study “veteran” criteria. As Multimedia Appendix 5 indicates, over half of the participants identified as white, followed by Hispanic, African American, Asian, Native American, and Hawaiian/Pacific Islander. Regarding education, just over 10% of the sample had a high school diploma/general equivalency diploma or less; the largest group had attended some college or trade school; others had completed an Associate or other 2-year degree. The remainder had completed a Bachelor degree or more, with a noticeable number of participants having either received a graduate degree or completed some graduate work. The vast majority of veterans were male (147/181, 81.2%) and the vast majority of partners (129/139, 92.8%) were female.

#### Service History

The majority of veterans in this study served in the Army (102/181, 56.4%), followed by the Marine Corps (43/181, 23.8%), the Navy (23/181, 12.7%), and the Air Force (13/181, 7.2%). At the time the study launched, the bulk of veterans had retired or separated from the service (123/181, 68.0%) and the remainder were still serving (58/181, 32.0%). On average, these veterans had served 2 deployments. The most frequent year of first deployment was 2005, and of last return, 2008.

### Fidelity (Use of MR)

There were no absolute requirements for use of MR practices other than to both give and receive one 20-minute massage per week for the first 8 weeks. We wanted participants in the MR arms to use the range of nonmassage techniques offered in the way they felt was most efficacious for them, knowing that this could vary widely. We were interested to learn which practices participants chose to use most frequently. Thus, we requested that all participants in both MR arms try every practice at least once early on, then use whatever practices they felt helped them most, as often as they wished.

With a complex and self-directed program such as MR, use, or fidelity, is a critical question. If we build it, will they use it? And will those who use it more derive more benefit? With these questions in mind, we tracked use through their weekly reports.

As Table 2 indicates, during weeks 1–8, on average veterans and partners used some aspect of MR 20 times per week, totaling nearly 2.5 hours per week. With an average of more than 17 “uses” of nonmassage practices per week by both veterans and partners, and an average of 1.4 massages per week received by both veterans and partners, the usage data met and surpassed our hypothesized use of the program (hypotheses 2 and 3). The most frequently used practice cluster was *connecting with yourself*, with morning gratitude being most popular, followed by the mirror greeting. Since this entire cluster involves relatively brief practices, frequency does not correlate with most time spent. Nearly twice as much time was spent in *connecting with quiet* and even more than that in *connecting with your partner*. The 2 massages each averaged roughly 20 minutes and the contemplative exercise of seeing each other with fresh eyes averaged nearly 15 minutes. The large standard deviations in this table indicate widely differing usage patterns. Cumulative totals for each cluster indicate that both veterans and partners spent over half the MR time per week in exercises to enhance connection with their partner. (See Multimedia Appendix 6 for MR usage reports for weeks 2, 8, and 16.)

Weeks 9–16 required no weekly reports, but the final survey at T3 included the weekly report questions answered just for week 16, to collect data on any longitudinal pattern changes. Frequency of activity use had decreased somewhat in all 3 MR clusters for veterans, as had time spent, shrinking a bit, yet still remaining above 90 minutes of weekly use on average. Partners, on the other hand, increased their mean total time per week by over 10 minutes, due entirely to increased use of the *connecting with yourself* and *connecting with quiet* practices.

**Table 2:** Weekly Mission Reconnect use over the 8-week monitoring period (n=79 dyads of veterans and their partners).

Component		Veterans (692 reports)		Partners (559 reports)	
		Frequency, mean (SD)	Minutes, mean (SD)	Frequency, mean (SD)	Minutes, mean (SD)
**Connecting with yourself**					
	Loosening and relaxing	1.9 (1.9)	12.8 (23.3)	1.8 (2.2)	11.8 (19.9)
	Waking up the body	1.9 (2.1)	12.0 (23.5)	1.7 (2.2)	12.3 (25.6)
	Reset and refresh	2.2 (3.1)	N/A^a^	2.6 (3.4)	N/A
	Morning gratitude	2.6 (2.2)	N/A	3.1 (2.4)	N/A
	Mirror greeting	2.5 (2.9)	N/A	2.5 (2.5)	N/A
	Cumulative subtotals	11.1 (9.6)	24.8 (44.8)	11.7 (10.2)	24.2 (43.6)
**Connecting with quiet**					
	Centering	2.1 (2.3)	16.3 (27.1)	2.3 (2.8)	17.7 (27.4)
	Movement into stillness	1.3 (1.6)	8.5 (15.5)	1.3 (2.1)	11.7 (26.6)
	Deep relaxation	1.7 (1.9)	22.5 (39.0)	1.5 (2.1)	17.3 (30.1)
	Cumulative subtotals	5.0 (4.9)	47.3 (65.7)	5.2 (6.0)	46.7 (76.1)
**Connecting with your partner**					
	Seeing each other	1.2 (1.7)	14.1 (35.0)	1.4 (2.0)	15.8 (34.4)
	Giving massage to your partner	1.4 (1.8)	29.0 (38.3)	1.4 (2.3)	29.0 (88.5)
	Receiving massage from your partner	1.4 (1.8)	23.9 (53.6)	1.4 (2.3)	28.8 (88.3)
	Cumulative subtotals	4.1 (4.4)	72.5 (103.3)	4.0 (5.7)	73.2 (186.3)
Totals for all activities		20.2 (15.1)	144.6 (164.8)	20.5 (18.3)	142.2 (244.0)

^a^N/A: not applicable or not available. Since these practices take very little time, often less than one minute, we asked subjects to report frequency only, not minutes spent.

### Non-MR Participants’ Wellness Strategies

Participants in the PREP-alone and waitlist arms employed a range of techniques to help themselves relax and to support their general well-being, ease pain or tension, support their partner and strengthen their relationship, or improve their sleep. These included prescription and nonprescription drugs, sex, meditation, exercise, baths, yoga, and reading. Both veterans and partners reported most frequently engaging in activities for relaxation or well-being, followed by support for the relationship or partner, and easing pain or tension, and least frequently for improving sleep. Veterans averaged 3.6 total activities per week and partners 3.9 (see Multimedia Appendix 7 for a description of activities reported).

### Survey Data: Veterans

While we assigned 40 dyads to each arm, for dual-veteran dyads we analyzed both participants as veterans, leading to sample sizes larger than 40. Table 3 presents veterans’ scores by arm at all 3 testing points. Baseline differences between arms were few and relatively modest except a PainBest difference between arms 1 and 3. *P* values shown reflect significant differences for T2 and T3 relative to baseline scores.

The most striking differences were between the MR-only and waitlist control arms. The MR-only veterans had improvements at 8 weeks in a broad array of mental health dimensions, indeed, everything other than pain scores, dyadic adjustment scores, and perceived social support. All improvements were sustained at 16 weeks with the exception of sleep improvement (PSQItotal). Perceived ability to respond to stressful events (RSES) had further improved.

The PREP-only veterans (arm 3) showed a modest gain in three important dimensions at T2 (PSS, BDI, and PCL-C), which became stronger by T3. The MR+PREP arm was included to see whether there would be added value by combining the programs. These results indicate that adding MR to PREP produces improvement in more domains than PREP alone (adding improvements in self-compassion and capacity to respond to stress). However, the combination yielded a lower magnitude of improvement in those arenas than resulted from MR alone. Thus, aim 1, our hypothesis that veterans and partners assigned to MR would report significantly greater improvements in a wide range of mental health outcomes, was partially met in that veterans in the MR-only arm reported significant improvements for a broader array of mental health outcomes than veterans in the other arms.

#### PTSD Subanalysis

The data indicate significant reductions, of varying degrees, in PCL-C scores for veterans in all 3 intervention arms. To assess outcomes for veterans entering with high levels of PTSD, we additionally analyzed those with baseline scores ≥50, which is commonly considered a clinical diagnostic criterion. This subset included 53/181 (29.3%) veterans in the sample. As seen in Table 4, high scorers in the MR-only arm had clinically significant reductions, dropping them to below the diagnostic threshold at both follow-ups. Their peers in the MR+PREP and PREP-alone arms also showed significant reductions, though of lesser magnitude.

**Table 3:** Veterans’ within-group changes from baseline to 8- and 16-week follow-ups (paired *t* tests).

Scale		Arm 1: MR^a^			Arm 2: MR+PREP^b^			Arm 3: PREP			Arm 4: waitlist		
		n	Mean (SD)	*P* value	n	Mean (SD)	*P* value	n	Mean (SD)	*P* value	n	Mean (SD)	*P* value
**Baseline (T1)**																
	PSS^c^	45	20.0 (6.7)		44	19.2 (6.3)		43	21.1 (6.4)		48	20.5 (6.5)	
	BDI^d^	45	14.5 (10.8)		44	16.1 (12.5)		43	19.5 (12.0)		48	16.8 (12.7)	
	PCL-C^e^	45	38.4 (16.5)		44	41.7 (18.3)		43	42.1 (16.1)		48	41.1 (15.8)	
	SCStotal^f^	45	73.9 (20.8)		44	72.6 (21.9)		44	73.2 (22.0		48	72.3 (18.8)	
	RSES^g^	45	57.8 (14.1)		44	55.3 (19.7)		44	50.9 (20.5)		48	54.7 (15.3)	
	MSPSStotal^h^	45	60.2 (18.3)		44	62.1 (15.2)		44	59.8 (17.1)		48	62.2 (15.6)	
	PSQItotal^i^	45	9.2 (4.2)		44	11.2 (4.4)		44	10.7 (4.0)		48	10.5 (4.1)	
	RDAS^j^	45	46.1 (10.2)		44	47.0 (9.6)		44	42.7 (11.5)		48	43.1 (9.9)	
	PainUsual^k^	45	3.1 (2.6)		44	4.5 (2.7)		44	4.5 (2.9)		48	4.2 (2.6)	
	PainBest^l^	45	1.6 (1.8)		44	2.5 (2.3)		44	2.7 (2.1)		48	2.1 (2.0)	
**8 weeks (T2)**																
	PSS	44	15.5 (7.2)	.0001*	42	16.2 (7.3)	.007	43	19.2(6.6)	.03	47	18.4 (7.2)	.002*
	BDI	43	9.4 (12.3)	.0004*	42	10.9 (10.5)	.002*	40	14.8 (12.4)	.004*	45	15.0 (13.1)	.43
	PCL-C	44	32.3 (15.8)	.0002*	42	35.0 (16.2)	.006	42	37.9 (16.7)	.02	48	39.6 (15.8)	.15
	SCStotal	44	85.6 (22.5)	.0001*	43	77.5 (18.8)	.09	39	77.2 (21.3)	.37	45	74.8 (18.4)	.32
	RSES	44	64.6 (15.6)	.002*	43	57.7 (18.7)	.31	42	53.0 (22.3)	.43	47	55.1 (16.6)	.57
	MSPSStotal	43	62.5 (17.7)	.44	42	65.9 (14.4)	.17	42	61.0 (17.0)	.64	47	61.9 (15.7)	.77
	PSQItotal	44	7.6 (4.1)	.003*	42	10.2 (4.7)	.07	42	10.8 (4.3)	.62	48	10.0 (4.5)	.25
	RDAS	43	47.1 (11.0)	.64	40	50.7 (9.8)	.03	42	42.8 (11.9)	.98	46	43.0 (10.5)	.56
	PainUsual	44	3.4 (2.8)	.56	43	4.0 (2.8)	.25	41	4.0 (2.7)	.25	48	4.2 (2.6)	.95
	PainBest	44	1.8 (2.3)	.55	43	2.3 (2.2)	.72	41	2.2 (2.1)	.09	48	2.2 (2.0)	.57
**16 weeks (T3)**																
	PSS	44	15.0 (7.3)	.0001*	43	16.2 (7.8)	.009	42	18.2 (5.6)	.0001*	48	19.5 (7.2)	.27
	BDI	44	8.7 (12.5)	.0003*	42	12.4 (14.7)	.04	41	13.9 (11.8)	.0009*	46	14.7 (13.1)	.99
	PCL-C	44	31.3 (15.7)	.0002*	42	36.1 (18.7)	.01	42	35.1 (14.1)	.0004*	46	39.4 (17.3)	.77
	SCStotal	43	87.7 (24.4)	.0001*	43	82.3 (2.2)	.004*	42	76.7 (22.3)	.12	47	73.4 (2.2)	.63
	RSES	43	65.3 (17.6)	.0006*	42	62.0 (2.1)	.007	42	54.5 (18.0)	.06	48	56.4 (17.3)	.59
	MSPSStotal	43	64.9 (16.9)	.08	43	63.0 (18.1)	.99	42	62.5 (15.2)	.32	47	62.0 (18.2)	.49
	PSQItotal	44	8.2 (5.0)	.08	41	10.7 (4.8)	.45	41	10.0 (4.5)	.21	47	10.7 (4.5)	.635
	RDAS	44	47.9 (1.9)	.21	39	49.6 (11.3)	.27	42	42.5 (11.6)	.67	46	43.9 (9.4)	.857
	PainUsual	44	2.7 (2.4)	.11	42	4.3 (3.1)	.66	41	4.2 (3.0)	.44	46	4.5 (2.7)	.426
	PainBest	44	1.4 (1.9)	.37	42	2.5 (2.5)	.47	41	2.6 (2.2)	.63	46	2.5 (2.3)	.362

^a^MR: Mission Reconnect.

^b^PREP: Prevention and Relationship Enhancement Program.

^c^PSS: Perceived Stress Scale-10 item.

^d^BDI: Beck Depression Inventory.

^e^PCL-C: PTSD Checklist-Civilian version.

^f^SCStotal: Self-Compassion Scale.

^g^RSES: Response to Stressful Experiences Scale.

^h^MSPSStotal: Multidimensional Scale of Perceived Social Support.

^i^PSQItotal: Pittsburgh Sleep Quality Index.

^j^RDAS: Revised Dyadic Adjustment Scale.

^k^PainUsual: rating of usual pain level over the past week.

^l^PainBest: rating of best pain level over the past week.

*P<.05 after adjustment for experimentwise error rate (true *P* values before adjustment displayed).

**Table 4:** Changes at 8 (T2) and 16 weeks (T3) in PTSD Checklist-Civilian version (PCL-C) scores for veterans with baseline (T1) score ≥50 (paired *t* tests)

Arm	n	T1 scores	T1 vs T2 scores				T1 vs T3 scores			
		Mean (SD)	Mean change	*t*	*df*	*P* value	Mean change	*t*	*df*	*P* value
MR^a^	12	61.2 (10.0)	–12.2	–4.6	11	.001*	–13.5	–3.9	11	.003*
MR+PREP^b^	14	63.9 (9.7)	–10.6	–2.2	13	.048	–5.6	–1.4	13	.18
PREP	13	62.8 (8.3)	–7.9	–2.3	12	.046*	–12.1	–3.5	12	.006*
Waitlist	14	60.6 (7.0)	–3.7	–1.9	13	.08	–1.2	–.5	13	.66

^a^MR: Mission Reconnect.

^b^PREP: Prevention and Relationship Enhancement Program.

*P<.05 after adjustment for experimentwise error rate (true *P* values before adjustment displayed).

#### Group Comparisons

Table 5 presents results of 2-sample *t* tests at 8 weeks, indicating no significant differences between the 2 non-MR arms at that point. However, there were some notable differences between MR-only and each of the other arms, particularly regarding the capacity to respond to stress and quality of sleep during the first 8 weeks. Table 6 shows results at T3, indicating that contrasts had strengthened, with more dimensions reaching significant differences by 16 weeks. These tables indicate that the number of mental health outcomes for which difference in magnitude of improvement was significant for MR compared with the other groups was limited and did not match our expectations as stated in hypothesis 1.

**Table 5:** Veterans’ between-group differences at 8 weeks (T2) (2-sample *t* tests).

Scale	Arm comparisons (*P* values)					
	MR^a^ vs MR+PREP^b^	MR vs PREP	MR vs waitlist	MR+PREP vs PREP	MR+PREP vs waitlist	PREP vs waitlist
PSS^c^	.66	.02	.07	.05	.17	.56
BDI^d^	.56	.054	.04	.13	.12	.92
PCL-C^e^	.44	.12	.03	.43	.18	.61
SCStotal^f^	.07	.09	.02	.95	.49	.57
RSES^g^	.06	.007*	.006*	.30	.49	.62
MSPSStotal^h^	.34	.69	.87	.16	.22	.79
PSQItotal^i^	.008*	.001*	.01*	.58	.82	.42
RDAS^j^	.12	.09	.08	.002*	.001*	.94
PainUsual^k^	.31	.27	.16	.94	.74	.80
PainBest^l^	.35	.41	.32	.90	.99	.90

^a^MR: Mission Reconnect.

^b^PREP: Prevention and Relationship Enhancement Program.

^c^PSS: Perceived Stress Scale-10 item.

^d^BDI: Beck Depression Inventory.

^e^PCL-C: PTSD Checklist-Civilian version.

^f^SCStotal: Self-Compassion Scale.

^g^RSES: Response to Stressful Experiences Scale.

^h^MSPSStotal: Multidimensional Scale of Perceived Social Support.

^i^PSQItotal: Pittsburgh Sleep Quality Index.

^j^RDAS: Revised Dyadic Adjustment Scale.

^k^PainUsual: rating of usual pain level over the past week.

^l^PainBest: rating of best pain level over the past week.

*P<.05 after adjustment for experimentwise error rate (true *P* values before adjustment displayed).

**Table 6:** Veterans’ between-group differences at 16 weeks (T3) (2-sample *t* tests).

Scale	Arm comparisons (*P* values)					
	MR^a^ vs MR+PREP^b^	MR vs PREP	MR vs waitlist	MR+PREP vs PREP	MR+PREP vs waitlist	PREP vs waitlist
PSS^c^	.44	.03	.004*	.19	.04	.33
BDI^d^	.22	.054	.03	.61	.43	.76
PCL-C^e^	.20	.24	.02	.78	.40	.21
SCStotal^f^	.27	.03	.003*	.23	.04	.48
RSES^g^	.42	.006*	.02	.07	.16	.60
MSPSStotal^h^	.61	.49	.43	.90	.80	.89
PSQItotal^i^	.02	.09	.01	.48	.99	.45
RDAS^j^	.49	.03	.07	.007*	.01	.51
PainUsual^k^	.01*	.01	.002*	.89	.76	.64
PainBest^l^	.02	.008	.02	.87	.96	.82

^a^MR: Mission Reconnect.

^b^PREP: Prevention and Relationship Enhancement Program.

^c^PSS: Perceived Stress Scale-10 item.

^d^BDI: Beck Depression Inventory.

^e^PCL-C: PTSD Checklist-Civilian version.

^f^SCStotal: Self-Compassion Scale.

^g^RSES: Response to Stressful Experiences Scale.

^h^MSPSStotal: Multidimensional Scale of Perceived Social Support.

^i^PSQItotal: Pittsburgh Sleep Quality Index.

^j^RDAS: Revised Dyadic Adjustment Scale.

^k^PainUsual: rating of usual pain level over the past week.

^l^PainBest: rating of best pain level over the past week.

*P<.05 after adjustment for experimentwise error rate (true *P* values before adjustment displayed).

### Survey Data: Partners

The 4 partner arms were also quite comparable at baseline (Table 7). Differences were that arm 1 had higher perceived stress than arm 4, and arm 3 differed from arms 2 and 4 on the PainBest and PainUsual scores. However, partners differed from veterans in terms of program outcomes.

Examining change within each arm from baseline to T2 and T3, we see no significant changes for either of the non-MR arms. The MR-only arm shows change in the same arenas for partners as for veterans, but a bit less powerfully at T2 and somewhat diminished at T3. For MR+PREP, the changes for partners strengthen from T2 to T3, which may reflect partners’ increased use of the program in weeks 8–16, when veterans had decreased both duration and frequency of use. Despite this within-group change for the MR arms, there were no statistically significant differences between the 4 arms as time progressed, other than for pain.

Group differences for partners at T2 and T3 were not noteworthy and are not reported here (available upon request).

**Table 7:** Partners’ within-group changes from baseline to 8- and 16-week follow-ups (paired *t* tests), as measured by standardized instruments.

Scale		Arm 1: MR^a^			Arm 2: MR+PREP^b^			Arm 3: PREP			Arm 4: waitlist		
		n	Mean (SD)	*P* value	n	Mean (SD)	*P* value	n	Mean (SD)	*P* value	n	Mean (SD)	*P* value
**Baseline (T1)**													
	PSS^c^	35	20.2 (5.7)		36	18.3 (6.5)		36	17.5 (7.2)		32	17.0 (5.7)	
	BDI^d^	35	13.6 (9.7)		36	13.3 (1.4)		36	10.9 (1.9)		32	9.9 (8.1)	
	PCL-C^e^	35	33.7 (12.6)		36	33.4 (15.0)		36	29.2 (11.7)		32	29.1 (9.0)	
	SCStotal^f^	35	81.7 (18.5)		36	77.0 (2.5)		36	85.1 (23.1)		32	84.3 (16.4)	
	RSES^g^	35	62.9 (14.6)		36	59.0 (19.5)		36	63.0 (17.8)		32	62.8 (16.0)	
	MSPSStotal^h^	35	63.6 (15.2)		36	65.4 (18.2)		36	66.2 (13.7)		32	67.3 (11.3)	
	PSQItotal^i^	35	9.1 (4.5)		36	9.0 (4.2)		36	7.8 (4.3)		32	8.0 (4.3)	
	RDAS^j^	35	45.4 (12.5)		36	46.4 (11.7)		36	44.8 (1.5)		32	44.7 (8.7)	
	PainUsual^k^	35	2.9 (2.8)		36	3.9 (2.0)		36	2.5 (2.2)		32	3.4 (2.5)	
	PainBest^l^	35	1.4 (1.8)		36	1.8 (1.9)		36	.9 (1.2)		32	1.6 (1.7)	
**8 Weeks (T2)**																
	PSS	34	16.3 (6.2)	.004*	35	16.5 (7.0)	.02	35	16.2 (6.9)	.17	31	18.1 (5.8)	.27
	BDI	34	7.6 (9.9)	.0001*	35	10.0 (11.2)	.02	36	10.2 (11.1)	.70	31	10.3 (1.2)	.99
	PCL-C	32	29.1 (12.7)	.0005*	33	30.7 (14.3)	.04	36	30.3 (14.5)	.67	32	29.6 (1.2)	.77
	SCStotal	33	90.2 (19.6)	.01	33	83.5 (21.6)	.006	35	87.4 (22.3)	.25	31	84.7 (18.9)	.63
	RSES	34	65.7 (13.7)	.35	35	61.2 (2.5)	.23	34	61.4 (19.0)	.65	32	61.7 (17.8)	.59
	MSPSStotal	34	68.2 (12.7)	.01	33	65.4 (2.6)	.96	36	68.1 (15.2)	.25	31	66.3 (11.9)	.49
	PSQItotal	34	6.4 (4.2)	.0002*	34	7.9 (4.6)	.17	36	7.4 (4.1)	.44	31	8.4 (5.2)	.64
	RDAS	34	46.8 (13.2)	.31	35	47.7 (12.3)	.39	36	44.5 (11.5)	.88	32	44.6 (1.3)	.86
	PainUsual	34	3.1 (2.4)	.64	34	3.1 (2.8)	.04	36	2.4 (2.4)	.78	32	3.7 (2.6)	.43
	PainBest	34	1.2 (1.8)	.67	34	1.6 (2.4)	.80	36	.9 (1.3)	.92	32	1.9 (2.2)	.36
**16 Weeks (T3)**																
	PSS	33	15.6 (7.0)	.002*	35	15.1 (7.1)	.002*	35	16.0 (7.7)	.13	32	17.6 (7.1)	.48
	BDI	33	9.3 (11.1)	.02	32	8.2 (1.0)	.002*	36	10.3 (1.3)	.68	32	10.8 (9.1)	.51
	PCL-C	34	30.3 (15.1)	.09	34	28.7 (14.4)	.007*	35	29.1 (14.6)	.66	32	29.9 (12.0)	.65
	SCStotal	33	91.1 (21.2)	.003*	32	87.5 (2.8)	.001*	33	88.0 (22.3)	.21	32	86.6 (2.4)	.22
	RSES	31	65.2 (13.1)	.17	34	62.5 (2.9)	.104	34	62.1 (18.9)	.88	32	61.4 (16.6)	.40
	MSPSStotal	34	65.0 (16.3)	.63	32	62.1 (22.9)	.33	34	67.9 (11.3)	.48	32	64.3 (14.3)	.22
	PSQItotal	33	7.4 (4.5)	.01	34	7.3 (4.4)	.003*	34	7.9 (4.5)	.28	32	8.8 (4.4)	.24
	RDAS	33	47.3 (12.3)	.19	33	47.8 (13.5)	.77	34	44.6 (1.7)	.64	32	44.2 (1.8)	.60
	PainUsual	32	2.5 (2.6)	.20	34	2.7 (2.8)	.03	34	2.3 (2.2)	.73	32	3.7 (3.1)	.53
	PainBest	32	1.2 (2.0)	.54	34	1.3 (2.0)	.09	34	1.4 (1.9)	.05	32	2.1 (2.2)	.29

^a^MR: Mission Reconnect.

^b^PREP: Prevention and Relationship Enhancement Program.

^c^PSS: Perceived Stress Scale-10 item.

^d^BDI: Beck Depression Inventory.

^e^PCL-C: PTSD Checklist-Civilian version.

^f^SCStotal: Self-Compassion Scale.

^g^RSES: Response to Stressful Experiences Scale.

^h^MSPSStotal: Multidimensional Scale of Perceived Social Support.

^i^PSQItotal: Pittsburgh Sleep Quality Index.

^j^RDAS: Revised Dyadic Adjustment Scale.

^k^PainUsual: rating of usual pain level over the past week.

^l^PainBest: rating of best pain level over the past week.

*P<.05 after adjustment for experimentwise error rate (true *P* values before adjustment displayed).

### Massage Effects

We included partner massage as an important element of MR both to give couples another way to communicate their care and because our own prior studies found that brief massage by family members significantly reduced troublesome symptoms [23,34]. Both veterans and partners reported highly significant reductions in all assessed symptoms of physical pain, tension, irritability, anxiety or worry, and depression shortly after receiving massage (Table 8), thus supporting hypothesis 4. In addition, both veterans and partners reported significant reductions in most premassage symptoms over the 8-week period (Table 9). While the short-term improvements recorded in the pre- and postmassage scores can reasonably be attributed to massage effects, the premassage changes over time cannot. They may be influenced, at least in part, by the MR program use overall, but we did not directly test that.

**Table 8:** Massage session effects on veteran-partner dyads: changes in symptom ratings (Wilcoxon signed rank tests).

Symptoms		Before mean (SD)	After mean (SD)	S^a^	*P* value
**Veterans (n=453)**					
	Physical pain	3.6 (2.6)	2.1 (2.0)	26555	.0001*
	Physical tension	4.2 (2.3)	1.9 (1.8)	40798.5	.0001*
	On edge/irritable	3.8 (2.7)	1.6 (1.9)	34376	.0001*
	Anxiety/worry	3.7 (2.8)	1.7 (2.1)	31278	.0001*
	Depression	2.3 (2.7)	1.2 (1.9)	13169.5	.0001*
**Partners (n=371)**					
	Physical pain	3.6 (2.6)	2.1 (2.1)	20518	.0001*
	Physical tension	4.5 (2.5)	2.0 (1.9)	28796	.0001*
	On edge/irritable	4.0 (2.7)	1.6 (1.9)	26398	.0001*
	Anxiety/worry	4.1 (2.7)	1.9 (2.1)	23646.5	.0001*
	Depression	2.3 (2.7)	1.4 (2.1)	6310.5	.0001*

^a^Signed rank statistic.

*P<.05 after adjustment for experimentwise error rate (true *P* values before adjustment displayed).

**Table 9:** Changes in premassage symptom ratings over 8 weeks in veteran-partner dyads (Kendall tau-b results).

Symptoms		τ	*P* value
**Veterans (453 session reports)**			
	Physical pain	–.003	.92
	Physical tension	–.116	.001*
	On edge/irritable	–.113	.002*
	Anxiety/worry	–.122	.0005*
	Depression	–.055	.13
**Partners (371 session reports)**			
	Physical pain	–.019	.63
	Physical tension	–.096	.01
	On Edge/irritable	–.089	.02
	Anxiety/worry	–.112	.004*
	Depression	–.088	.03

*P<.05 after adjustment for experimentwise error rate (true *P* values before adjustment displayed).

### Power

In view of the encouraging phase I feasibility results [23], after adding a margin of conservatism, we chose a moderately large effect size, corresponding to a value of 0.7 for Cohen *d*. The analysis of veterans’ within-group change (Table 3), assuming n=40 per arm, and type I error probability alpha=.05, obtained a power of 1–beta=99%. Between-group changes (Table 5,Table 6) yielded a power of 87%. Partners’ within-group changes (Table 7), with n=32 per arm, obtained a power of 96%. Massage session effects (Table 8) yielded a power >99% for both veterans and partners.

### User Satisfaction

On the final survey (T3) we asked MR participants how likely they would be to recommend the program to a friend. On a 0–10 scale, veterans’ mean score was 8.7, and partners’ mean was 9.1, indicating high user satisfaction with the program.

## Discussion

### Key Findings

Our primary intention in designing MR was to offer veterans and their partners a flexible form of instruction in simple ways to improve their own well-being. The range of outcomes in which users had significant improvement can be seen as confirmation that MR teaches skills that improved their well-being. The fact that improvements, including reduced PTSD symptoms, and increased self-compassion, were sustained at the 16-week follow-up is particularly promising.

Hypothesis 1 was generally supported by the findings that MR participants had significant improvement on far more mental health outcomes than did participants in other arms of the study. The exceptions in this case were sleep quality (partners using MR had more stable improvements than veterans), and lack of movement in dyadic adjustment and perceived social support. We speculate that the lack of change in relationship variables may have been due to high baseline levels, which may have led to the decision to participate, but this is conjecture and warrants further study. Hypothesis 1 was not fully supported in that, while the 2-sample *t* tests indicated significantly stronger improvement among MR participants on many mental health outcomes, only a portion of these remained following adjustment for potential experimentwise error. The remaining hypotheses were strongly supported.

It is notable that both veterans and partners in the MR arms used this self-directed program for over 2 hours per week during the initial 8 weeks, surpassing our hypothesized use, and averaged well over an hour per week throughout the 16-week data collection period. We suspect the control each user had over which program elements to use and when contributed to the high use level, underscoring the value of user preference in long-term adherence to, and ensuing effectiveness of, self-care approaches for this population.

Sustained use may also have been enhanced through the inherent support and encouragement of compliance by coparticipation with a significant relationship partner. We note, however, that the majority of MR practices (except for massage) can be used either alone or with others. This adds to the convenience for young parents, allowing one partner to practice while the other attends to their children.

In comparing the trial’s 4 arms, the contrast for both veterans and partners is greatest between MR-only and waitlist control arms, a predictable finding based on phase I results. The contrast in outcomes for the MR-only versus PREP arms, especially at 8 weeks, is notable. While PREP has been found in multiple studies to support significant improvement in variables related to relationship dynamics and intimacy for couples, we chose outcome measures to assess other dimensions of mental health. We selected some MR components based on evidence of health-promoting neurological and neurochemical effects; potential easing of symptoms related to PTSD, stress, and depression; and likelihood of enhancing self-compassion and forgiveness. We expected the physical exercises to amplify energy, improve mood, and reduce susceptibility to the fight-flight-freeze response. While PREP offers some content aimed at relaxation, stress reduction, and forgiveness, it devotes more time to relationship skills per se than does MR.

Thus, one interpretation of the outcomes is that MR performed well at its goals and PREP does less well at the things MR is designed to do. As to whether combining the programs adds value, adding MR to PREP appears to amplify improvement in self-compassion and response to stressful events. Adding PREP did not enhance other results for MR-only.

The program showed strong benefit for both men and women veterans and for their partners, although benefits were strongest for veterans. Based on these findings, which included veterans from all branches of service, and participants both on active duty and retired or separated from service, it appears that the program provides a safe, low-cost self-care intervention that enhances overall well-being. A modified version could be useful for single veterans.

### Limitations

This study evaluated MR in a community-based sample with no inclusion or exclusion criteria related to specific mental or physical health parameters. Thus, the program’s impact in clinically defined populations remains to be assessed. Other limitations are that the follow-up period was limited to 16 weeks and that the program was tested only with dyads, not single veterans. In addition, all participants were required to attend an in-person launch meeting, and it is possible that this excluded potential applicants for whom such attendance was impossible for reasons of time, geography, or something else. Finally, the instructional program as tested did not include video closed captioning or verbatim transcripts of the audio instruction, and thus would not have accommodated users with hearing limitations. These enhancements will be considered in future upgrading.

### Suggested Research

Four directions for future research on MR for veterans present themselves. First is to test whether adherence and benefit are still present at the 1-year mark or even further with a partnered sample such as we used. Second is to offer MR, minus the partner massage, to nonpartnered veterans to see whether lack of a partner affects use or outcomes. Third, it is important to follow this study with research on diagnostically defined samples (eg, high PTSD) to see what benefit might be provided for a clinically based sample. Fourth, we also recommend that health services research be done to determine how best to offer MR. This study demonstrates that veteran-partner dyads can learn a range of physical and contemplative self-care practices on their own from a media program accessed via mobile app or webstream, and that they can derive great benefit across several dimensions of well-being. Still, it is possible that, especially for a clinically based or nonpartnered sample, use of the program would be enhanced by introduction through in-person group instruction, for example, with 1 to 4 structured sessions. While this would add cost to delivery of the program, it is possible that added benefit could justify the cost. Related to this, one could retest MR, forgoing the launch meetings and substituting Web-based instruction in the provision of weekly report and survey data.

In addition, we recognize that many nonveterans have PTSD, physical pain, lack of self-compassion, etc. MR, as it now exists, or with minor changes, could and should be tested with other samples. This could include others who have experienced severe trauma, such as refugees, youth and adults exposed to mass shootings such as that in an Orlando, FL nightclub in 2016, as well as people with chronic pain and more generalized anxiety.

### Conclusions

This study adds to the growing literature on the power of brief, repeated mind- and body-based practices to address physical, psychological, and spiritual well-being [50-52]. The results indicate that MR is a widely accessible, low-cost approach that supports well-being and reduces multiple symptoms among post-9/11 veterans and their partners. Both veterans and partners were able to learn and make sustained use of a range of wellness practices from a media-only source. While the launch meetings offered 90 minutes of human contact, they offered no instruction in any of the practices. These usage findings contrast with others’ findings of high dropout rates for clinic-based programs such as meditation instruction [53]. Home-based, self-directed interventions may be of particular service to veterans who are distant from, averse to, or prohibited by schedule from using professional services. The partner relationship may enhance sustained use of self-directed interventions for this population. Finally, these data suggest MR to be superior to waitlist and PREP for Strong Bonds in both within-group and between-group assessments. Notably, this distinction appears to increase over time.

## Appendix

Multimedia Appendix 1 Video Introduction to Mission Reconnect.

Multimedia Appendix 2 Weekly report cards for Mission Reconnect participants (MR Only and MR+PREP) during weeks 1-8.

Multimedia Appendix 3 Weekly report cards for non-Mission Reconnect participants (PREP+Waitlist) during weeks 1-8.

Multimedia Appendix 4 CONSORT eHealth checklist.

Multimedia Appendix 5 Table 2. Demographic Information.

Multimedia Appendix 6 Mission Reconnect program utilization at weeks 2, 8, and 16.

Multimedia Appendix 7 Wellness strategies of non-Mission Reconnect participants (PREP + Waitlist).

## Abbreviations

BDI: Beck Depression Inventory
DoD: Department of Defense
MR: Mission Reconnect
MSPSStotal: Multidimensional Scale of Perceived Social Support
PainBest: rating of best pain level over the past week
PainUsual: rating of usual pain level over the past week
PCL-C: PTSD Checklist-Civilian version
PREP: Prevention and Relationship Enhancement Program
PSQItotal: Pittsburgh Sleep Quality Index
PSS: Perceived Stress Scale-10 item
PTSD: posttraumatic stress disorder
RDAS: Revised Dyadic Adjustment Scale
RSES: Response to Stressful Experiences Scale
SCStotal: Self-Compassion Scale
VA: US Department of Veterans Affairs
