# Supplementary material for: Post-9/11 Veterans and Their Partners Improve Mental Health Outcomes with a Self-directed Mobile and Web-based Wellness Training Program: A Randomized Controlled Trial
Source: J Med Internet Res. 2016 Sep 27;18(9):e255. doi: 10.2196/jmir.5800 (PMC5059485; doi:10.2196/jmir.5800)
Supplement: Multimedia Appendix 2 [file jmir_v18i9e255_app2.pdf]

Use these forms during the week to track the number of times you use each activity for your **Weekly Report**.

Practice Tracking Form<br>Week of ___________							
1. Connecting with Yourself…	Su	Mo	Tu	We	Th	Fr	Sa
a. Loosening and Relaxing							
b. Waking up the Body							
c. Reset and Refresh							
d. Morning Gratitude							
e. Mirror Greeting							
2. Connecting with Quiet…	Su	Mo	Tu	We	Th	Fr	Sa
a. Centering							
b. Movement into Stillness							
c. Deep Relaxation							
3. Connecting with Your Partner…	Su	Mo	Tu	We	Th	Fr	Sa
a. Seeing Each Other							
b. Giving Massage to Your Partner							
c. Receiving Massage from Your Partner							
