# Supplementary material for: Post-9/11 Veterans and Their Partners Improve Mental Health Outcomes with a Self-directed Mobile and Web-based Wellness Training Program: A Randomized Controlled Trial
Source: J Med Internet Res. 2016 Sep 27;18(9):e255. doi: 10.2196/jmir.5800 (PMC5059485; doi:10.2196/jmir.5800)
Supplement: Multimedia Appendix 3 [file jmir_v18i9e255_app3.pdf]

Use these forms during the week to track the number of times you engage in any activity related to the purposes listed in 1, 2, 3, and 4 below. List up to three activities (a, b, and c) for each category of activity. This will help you complete your Weekly Report.

Activity Tracking Form

Week of ___________

1. Activities to relax, reduce stress and/or support your general wellbeing…	Su	Mo	Tu	We	Th	Fr	Sa
a.							
b.							
c.							
2. Activities to ease physical pain or tension…	Su	Mo	Tu	We	Th	Fr	Sa
a.							
b.							
c.							
3. Activities to support your partner or in some way strengthen your relationship…	Su	Mo	Tu	We	Th	Fr	Sa
a.							
b.							
c.							
4. Activities to improve sleep quality…	Su	Mo	Tu	We	Th	Fr	Sa
a.							
b.							
c.							
