# Supplementary material for: Post-9/11 Veterans and Their Partners Improve Mental Health Outcomes with a Self-directed Mobile and Web-based Wellness Training Program: A Randomized Controlled Trial
Source: J Med Internet Res. 2016 Sep 27;18(9):e255. doi: 10.2196/jmir.5800 (PMC5059485; doi:10.2196/jmir.5800)
Supplement: Multimedia Appendix 5 [file jmir_v18i9e255_app5.pdf]

(N=160 dyads)					
		Veterans (N=181)		Partners (N=139)*	
	Age: Mean (SD)	33.4 (6.6)		32.4 (SD 7.0)	
		N	%	N	%
Sex	Male	147	81.2	10	7.2
	Female	34	18.8	129	92.8
Race/ Ethnicity	White	95	52.5	71	51.1
	Hispanic	46	25.4	41	29.5
	Black	21	11.6	16	11.5
	Asian	9	5.0	7	5.0
	American Indian	7	3.9	2	1.4
	Hawaiian/Pacific	3	1.7	0	0.0
	Other	4	2.2	6	4.3
Education	Some high school	0	0.0	1	0.7
	HS grad or GED	20	11.0	12	8.6
	Some college/trade	69	38.1	35	25.2
	Assoc./2-yr degree	24	13.3	15	10.8
	Bachelors	35	19.3	36	25.9
	Some grad work	12	6.6	11	7.9
	Grad degree	21	11.6	29	20.9
*Includes 7 non-deployed post-9/11 Veterans and 4 of other eras.					
