# Supplementary figures and images for: Post-9/11 Veterans and Their Partners Improve Mental Health Outcomes with a Self-directed Mobile and Web-based Wellness Training Program: A Randomized Controlled Trial
Source: J Med Internet Res. 2016 Sep 27;18(9):e255. doi: 10.2196/jmir.5800 (PMC5059485; doi:10.2196/jmir.5800)

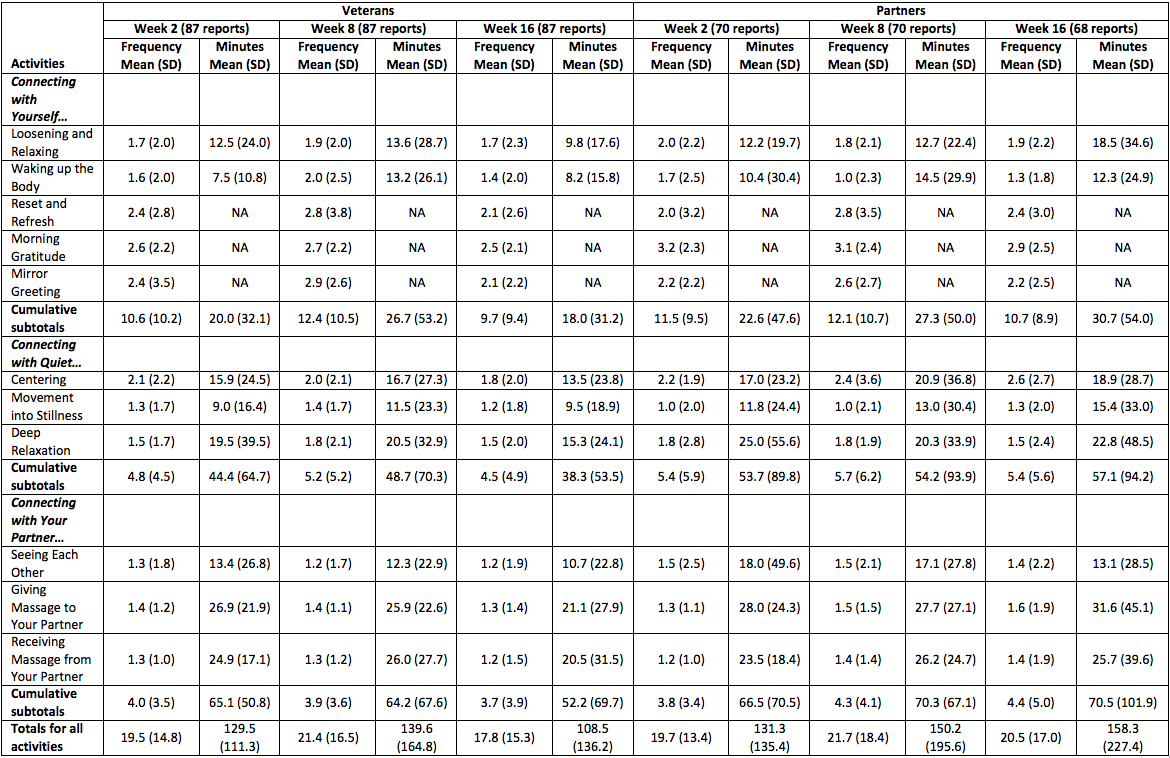

Supplement: Multimedia Appendix 6 [file jmir_v18i9e255_app6.png]

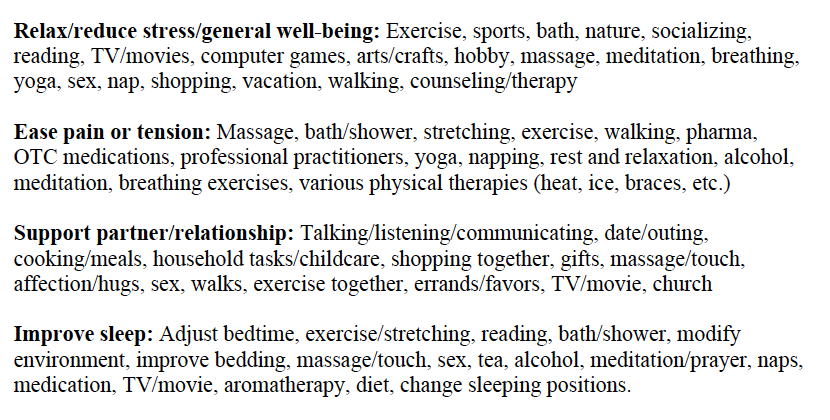

Supplement: Multimedia Appendix 7 [file jmir_v18i9e255_app7.png]
